# ‘Let him die in peace’: understanding caregiver’s refusal of medical oxygen treatment for children in Nigeria

**DOI:** 10.1136/bmjgh-2023-014902

**Published:** 2024-05-16

**Authors:** Ayobami Adebayo Bakare, Julius Salako, Carina King, Omotayo E Olojede, Damola Bakare, Olabisi Olasupo, Rochelle Burgess, Eric D McCollum, Tim Colbourn, Adegoke G Falade, Helle Molsted-Alvesson, Hamish R Graham, on behalf of the INSPIRING Project Consortium, Carina King

**Affiliations:** 1 Department of Global Public Health, Karolinska Institutet, Stockholm, Sweden; 2 Department of Community Medicine, University College Hospital, Ibadan, Nigeria; 3 Department of Paediatrics, University College Hospital, Ibadan, Nigeria; 4 Institute for Global Health, University College London, London, UK; 5 Global Program in Pediatric Respiratory Sciences, Eudowood Division of Pediatric Respiratory Sciences, Department of Pediatrics, School of Medicine, Johns Hopkins University, Baltimore, Maryland, USA; 6 Department of Paediatrics, University of Ibadan College of Medicine, Ibadan, Nigeria; 7 Centre for International Child Health, Murdoch Children’s Research Institute, University of Melbourne, MCRI, Royal Children's Hospital, Parkville, Victoria, Australia

**Keywords:** qualitative study, global health, health policy, child health

## Abstract

**Introduction:**

Efforts to improve oxygen access have focused mainly on the supply side, but it is important to understand demand barriers, such as oxygen refusal among caregivers. We therefore aimed to understand caregiver, community and healthcare provider (HCP) perspectives and experiences of medical oxygen treatments and how these shape oxygen acceptance among caregivers of sick children in Lagos and Jigawa states, which are two contrasting settings in Nigeria.

**Methods:**

Between April 2022 and January 2023, we conducted an exploratory qualitative study using reflexive thematic analysis, involving semistructured interviews with caregivers (Jigawa=18 and Lagos=7), HCPs (Jigawa=7 and Lagos=6) and community group discussions (Jigawa=4 and Lagos=5). We used an inductive-deductive approach to identify codes and themes through an iterative process using the theoretical framework of acceptability and the normalisation process theory as the analytic lens.

**Results:**

Medical oxygen prescription was associated with tension, characterised by fear of death, hopelessness about a child’s survival and financial distress. These were driven by community narratives around oxygen, past negative experiences and contextual differences between both settings. Caregiver acceptance of medical oxygen was a sense-making process from apprehension and scepticism about their child’s survival chances to positioning prescribed oxygen as an ‘appropriate’ or ‘needed’ intervention. Achieving this transition occurred through various means, such as trust in HCPs, a perceived sense of urgency for care, previous positive experience of oxygen use and a symbolic perception of oxygen as a technology. Misconceptions and pervasive negative narratives were acknowledged in Jigawa, while in Lagos, the cost was a major reason for oxygen refusal.

**Conclusion:**

Non-acceptance of medical oxygen treatment for sick children is modifiable in the Nigerian context, with the root causes of refusal being contextually specific. Therefore, a one-size-fits-all policy is unlikely to work. Financial constraints and community attitudes should be addressed in addition to improving client–provider interactions.

**WHAT IS ALREADY KNOWN**

**WHAT THIS STUDY ADDS**

- Medical oxygen treatment is an additional source of emotional distress for caregivers; therefore, caregivers of children prescribed medical oxygen therapy require support.
- Caregivers’ decision to accept oxygen treatment for sick children is a sense-making process.
- The financial implications of oxygen treatment for caregivers are a major reason for oxygen refusal in some contexts.
- The COVID-19 pandemic did not affect user’s perspectives on medical oxygen in Nigeria.

**HOW THIS STUDY MIGHT AFFECT RESEARCH, PRACTICE AND POLICY**

- Negative perceptions of oxygen are driven by modifiable contextual factors, with constructive provider–client interactions crucial for improved uptake of medical oxygen for children.
- Policies on medical oxygen access must not address only the supply side; making oxygen affordable to patients and hospitals should be the key priority of medical oxygen policies or strategic road maps.

## Introduction

 Medical oxygen is an essential commodity that remains inaccessible to many patients when needed, especially in low-income and middle-income countries (LMICs).^1^ Past efforts to address medical oxygen gaps in LMICs have focused mainly on improving the oxygen supply chain and accessibility through equipment and infrastructural support,^2^,^6^ technological innovation (eg, solar power and oxygen storage),^7^,^9^ capacity building for medical personnel^2 10 11^ and sustainable supply and distribution mechanisms.^12^ However, it is important to pay attention to patients’ and caregivers’ perceptions of medical oxygen treatment to improve demand and uptake when medical oxygen treatment is needed.

The 2023 World Health Assembly Resolution on medical oxygen access emphasised the importance of tackling oxygen acceptance among caregivers or patients, asking national governments to raise public awareness of the life-saving potential of oxygen treatment.^13^ In Nigeria, the 2017 National Oxygen Strategy included acceptance of medical oxygen treatment among patients and caregivers as a key objective.^14^

The under-five mortality rate remains disproportionately high in LMICs, with two-thirds of deaths caused by preterm birth complications, perinatal asphyxia, sepsis, pneumonia and malaria^15^—all conditions which commonly require medical oxygen treatment as part of treatment.^16^ However, there is evidence that medical oxygen treatment is not always accepted and is linked to several misconceptions.^17 18^ Previous studies on patient experience of medical oxygen and adherence are largely focused on adult populations and long-term oxygen use and rarely include motivation for oxygen therapy adherence or refusal, especially among the caregivers of young children.^19^,^24^

In a cross-sectional study conducted in Nigerian tertiary health facilities, 6.9% of caregivers of children admitted for care rejected medical oxygen treatment for their child, the most common reason being that oxygen therapy could kill their child. Education, knowledge and parity did not influence the acceptability of oxygen therapy, suggesting that community narratives of oxygen therapy may shape perceptions and acceptability of medical oxygen.^25^ Another study conducted in southwest Nigeria among adult patients and their caregivers reported that approximately 20% of the respondents believed that oxygen therapy was solely for terminally ill patients.^26^ Within other African settings, misconceptions about medical oxygen treatment and a lack of adequate information from healthcare providers (HCPs) were reported as barriers to oxygen acceptability among caregivers.^17 18 27^ Medical oxygen gained considerable global attention during the COVID-19 pandemic.^28 29^ However, studies on patient, community or HCP perceptions on medical oxygen remain scarce.

This study aimed to understand patient/caregiver, community and HCP perspectives and experiences of medical oxygen and explore how context and experience influence perception and acceptance of recommended oxygen treatment for sick children in Lagos and Jigawa states, which are two contrasting settings with different sociocultural and health system characteristics in Nigeria.

## Methods

### Study design

We conducted an exploratory qualitative study using reflexive thematic analysis, as described by Braun and Clark.^30^ Between April 2022 and January 2023, we conducted semistructured interviews with caregivers (Jigawa=18 and Lagos=7) and HCPs (Jigawa=7 and Lagos=6) and focus group discussions with community members (Jigawa=4 (8–9 community members per focus group) and Lagos=5 (12–24 community members per focus group)). The study was conducted as part of the process evaluation for the Integrated Sustainable childhood Pneumonia and Infectious disease Reduction in Nigeria (INSPIRING) project in Lagos and Jigawa states, which aimed to reduce under-five deaths from pneumonia through context-specific interventions.^10 31^ We followed the Consolidated Criteria for Reporting Qualitative Research guidelines.^32^

### Setting

The study was conducted in Kiyawa local government area (LGA), Jigawa state, northwest Nigeria and Ikorodu LGA, Lagos state, southwest Nigeria. These settings differ in terms of sociocultural and economic activities (see table 1).

**Table 1:** Characteristics of the study settings

Characteristics	Lagos	Jigawa
Setting	Urban	Rural and agrarian
Economy	Commercial and industrialised	Farming
Under-five mortality rate	15 per 1000 live births	174 per 1000 live births
Fertility rate	3.2	7.6
Health literacy	71.7	6.7
Predominant religion	Christianity and Islam	Islam
Predominant ethnic group	Yoruba	Hausa and Fulani

### Theoretical frameworks

The theoretical framework of acceptability informed the development of the topic guides (online supplemental appendix 1).^33^ Using the theoretical framework of acceptability, acceptability reflected the extent to which HCPs, patients, caregivers and community members considered medical oxygen treatment appropriate based on their anticipated or experienced cognitive and emotional response to the intervention.^33^ Given that acceptability is shaped by the broader social and political context,^34^,^36^ we used the socioecological model to guide questions on wider social-cultural norms and contextual factors like COVID-19 infection on oxygen acceptability.^37 38^ The data analysis was then informed by normalisation process theory—an action-based theory that focuses on how people make sense of complex interventions, such as medical oxygen, engage with them and reflect on their benefits.^39^ Normalisation process theory has been extensively used to evaluate healthcare interventions.^40^ The two theories complemented each other, with the theoretical framework of acceptability helping us identify initial manifest themes, while the coherence construct within the normalisation process theory helped us decipher the interconnectivity between them.

### Participants and sampling

Participants for caregiver and HCP interviews were identified in health facilities that routinely provide oxygen treatment services to sick children, including primary health facilities in Lagos.

For the caregiver interviews, purposive sampling was guided by maximum variation, and we aimed to include caregivers of children and neonates of different ethnic groups, levels of education and religions who had received oxygen, refused medical oxygen or had no indication of oxygen treatment but who received inpatient care at the time of the study. Study staff reviewed facility records and spoke to facility staff to identify admitted patients with characteristics of interest. Thereafter, caregivers were approached directly after obtaining permission from the ward and/or facility leaders.

For the HCP interviews, participants were approached directly by INSPIRING study staff in Lagos and Jigawa states. We purposively selected HCPs (doctors, nurses and community health workers) from different hospital units (emergency, inpatient and nursery) across different levels of healthcare delivery (table 2).

**Table 2:** Summary of participants’ characteristics

Healthcare providers		
Total	Lagos (n=6)	Jigawa (n=7)
Median age (range) years	35.5 (28–53)	35 (23–53)
Sex		
Female	5	3
Male	1	4
Profession		
Community health workers	0	1
Nurse/midwife	3	4
Medical doctor	3	1
Years of experience		
<10 years	3	3
≥10 years	3	4
Education		
Diploma	1	5
Bachelor’s degree	4	2
Master’s degree	1	0
Facility type		
Primary health centre	0	3
Secondary hospital	4	2
Tertiary hospital	0	2
Private hospital	2	0

Caregiver’s and child characteristics		
Total	Lagos (n=7)	Jigawa (n=18)
Child’s sex		
Female	2	8
Male	5	10
Child’s status at the time of interview		
Discharged alive	4	5
On admission	3	8
Dead	0	5
Received medical oxygen		
Yes	3	9
No	4	9
Median number of children ever born (range)	1 (1–4)	5 (2–16)
Median number of children alive (range)	1 (1–4)	3 (0–6)
Caregiver’s relationship		
Mother	5	18
Father	2	3
Grandfather	0	1
Caregiver’s religion		
Christianity	6	0
Islam	1	22

Focus group discussion		
Number of discussions	Lagos (n=5)	Jigawa (n=3)
Number of participants	50	25
Age (mean)	41.9 years	43.8 years
Education		
None, Arabic	3	10
Primary	9	2
Secondary	29	7
Tertiary	9	3
Religion		
Islam	27	25
Christianity	21	0
Traditional	1	0

For the community group discussions, participants were recruited through community mobilisers in Ikorodu and Kiyawa LGAs. The research team did not know or have any contact with the community participants prior to the day of the interview. Participants in the community group discussions included members of the wider caregiver networks, such as parents and grandparents. In Jigawa, we had separate discussions with men, older women and younger women. In Lagos, group discussions were separately conducted with men and women from areas typical of rural and urban contexts. Four caregivers declined participation in Lagos after the initial agreement, and none declined participation in Jigawa. In Lagos, we initially conducted four group discussions and later conducted one more due to pragmatic reasons to ensure high-quality data. No repeat interviews were conducted in either state.

### Data collection

Interviews were conducted in two phases, from 5 April 2022 to 29 January 2023, with a preliminary analysis conducted in between. After initial data familiarisation, we conducted six additional caregiver interviews in Jigawa to explore further emerging topics from the initial interviews. In Jigawa, interviews were conducted by AAB (a male medical doctor with experience in child health and oxygen systems and fluent in Yoruba and English languages) and two female-trained research nurses who are fluent in Hausa and English languages and have moderate experience in qualitative data collection. The Jigawa interviews were conducted in English or Hausa language. In Lagos, interviews were conducted by AAB and OEO, a male researcher fluent in Yoruba and English languages with a Masters of Public Health. OEO is familiar with the local setting and experienced in qualitative data collection. The Lagos interviews were conducted in English and Yoruba languages.

Among caregivers whose children had received oxygen, we explored their understanding of medical oxygen treatment, how this impacted their perceptions and acceptability and how they balanced their perceived risk of oxygen versus potential benefits. To minimise recall bias, interviews were conducted no longer than 4 weeks after discharge, referral or leaving against medical advice.

We used vignettes for the community group discussions. The vignettes focused on possible key recommendations by HCPs during acute illness episodes in children, such as referral, oxygen treatment, nasogastric tube feeding and antibiotic treatment. For discussion on oxygen treatment, we explored what they understood by the treatment, challenges families may face in accepting oxygen treatment, factors that may influence the decision to accept or refuse oxygen treatment and if the COVID-19 pandemic has affected their perception of medical oxygen.

For the HCP interviews, we explored their experience with oxygen therapy delivery for paediatric patients and challenges they had encountered in the process, such as non-acceptance by caregivers and how they handled it. Interviews with caregivers and HCPs were conducted in the health facility or participant’s place of residence, and nobody else was present besides the researchers and participants. Community group discussions were conducted in public spaces for community meetings (town hall and the residence of the community head). Caregiver and provider interviews lasted between 45 and 70 min, while community group discussions lasted between 80 and 150 min. We obtained audio-recordings and field notes for all interviews and discussions. The audio records were transcribed and translated into English and thereafter stored on a secure cloud server with restricted access. Participants were given US$3–4 Naira equivalent as incentives after the interview.

### Data analysis

The analysis team included AAB, CK, HRG and HMA. They are social and public health specialists from the INSPIRING team who are experts on medical oxygen at national and global levels.^2 10 30 41^ Reflexive thematic analysis was used throughout the analysis process.^30 42^ AAB first independently reviewed all transcripts for data familiarisation, writing notes on initial thoughts and feelings about the data. Next, the transcripts were reviewed to identify initial semantic codes. The codes were then collapsed into manifest themes, using the theoretical framework of acceptability as a guide.^33^ This entailed iterative reading of the transcripts and discussions with the analysis team to review the emerging themes. Following thoughtful engagement with the data, we discovered that oxygen acceptance was a spectrum and caregiver’s positions towards oxygen prescription may not be static. This fluidity was missing in the initial themes developed within the theoretical framework of acceptability. We therefore took a deeper dive into the data to achieve a richer interpretation, with a focus on the underlying processes driving the manifest theme as well as linking these processes together for a coherent story. At this stage, we used the ‘coherence’ construct of the normalisation process theory as the analytic lens to interpret the caregiver’s responses to the prescription of medical oxygen therapy and extract latent themes from the interview responses. These were further refined through the collaborative efforts of the analysis team in an iterative process. CK, HRG and HMA provided outsider’s reflections on the themes and AAB provided insider’s reflections on the data. In our context, we assumed medical oxygen prescriptions were unanticipated treatment by caregivers when they sought care for their children, unlike antimicrobials, which caregivers often demand during care-seeking episodes. We triangulated our findings between states and participant groups. We did not return transcripts to participants for review but shared our findings with them for feedback. The data were analysed using NVivo software.^43^

### Patient and public involvement

The overarching studies (the INSPIRING project in Lagos and Jigawa states) were designed through co-design workshops involving representatives from the Nigerian government, community-based organisations, HCPs, Save the Children and evaluation partners.^10 31^ However, patients were not involved in the design of this study, and findings from this study were not discussed with the public.

## Results

**Figure 1:**
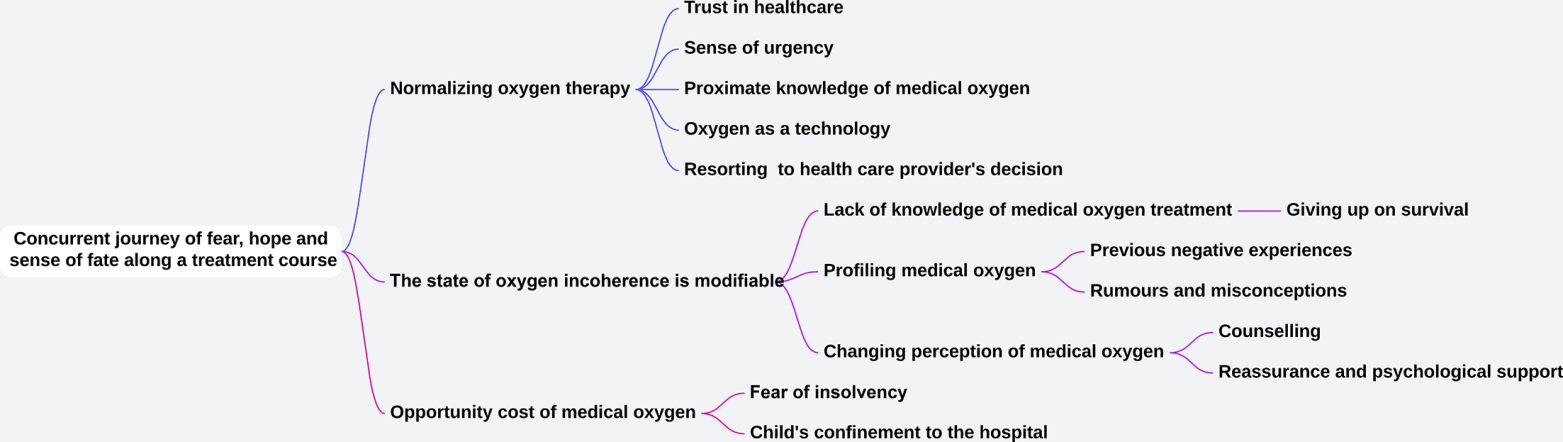
Coding tree from analysis of the transcripts

The overarching theme identified was ‘concurrent journeys of fear, hope and sense of fate along the treatment course’ (figure 1). This describes the struggles, fears and hopelessness experienced by caregivers following a medical oxygen prescription on the one hand, the feelings of hope and happiness with the successful application of medical oxygen treatment, and the sense of fate following a negative outcome in children who received medical oxygen.

In Lagos, where exposure and access to medical oxygen were higher, oxygen was generally considered a promising intervention, and the reason for oxygen refusal varied by participants. Community and HCPs highlighted cost and lack of awareness about the benefits of oxygen therapy as major barriers to oxygen acceptance, whereas poor communication from providers was an underlying factor for fear and reactions towards refusal in the caregiver interviews.

In Jigawa, where access and exposure to medical oxygen were limited, misconceptions about oxygen as a death sentence were common, but provider and caregiver interviews highlighted trust in HCPs and a perceived sense of urgency as overriding factors that favour caregiver acceptance of medical oxygen for sick children. In both Lagos and Jigawa, providing counselling and psychological support to caregivers was reported to improve oxygen acceptance by caregivers, and when it failed, care was sought from another source.

### Normalising oxygen therapy

Caregiver acceptance of oxygen treatment reflected a sense-making process that originates with apprehension and scepticism about the child’s survival chances following an oxygen prescription, positioning prescribed oxygen as an ‘appropriate’ or ‘needed’ intervention despite the mental, physical and financial burdens that caregivers encounter following a medical oxygen prescription for their children. Achieving this transition was triggered by various factors depending on geographical and social context and individual experiences, specifically trust in HCPs, a perceived sense of urgency for care, having proximate knowledge of medical oxygen treatment and a symbolic perception of oxygen as a technology.

Despite the lack of explanation from HCPs in both states regarding the need for medical oxygen, caregivers expressed moralistic and technical trust in HCPs. They believed that oxygen would not be administered if the HCP had not considered it beneficial to their child and were confident that oxygen would not harm the patient. This is reflected in the quotes below:

‘If the baby didn't need it, they won't give’. Caregiver 1 Lagos

‘The workers are out to help and protect their patients and not to harm. So, I knew it was the best he was doing for her (baby)’. Caregiver 14 Jigawa

In Jigawa, trust in treatments offered by HCPs was expressed as being based on relationships, earned through the perceived closeness of the providers to the community or the involvement of a trusted community member.

‘We are not from away. We are all from here, we were born here, we are relatives, so they are accepting (oxygen) because they know we are from them and we not going to cheat (harm) them’*.* Jigawa HCP 5

In Lagos, oxygen administration via oxygen concentrators was associated with fewer negative reactions from caregivers compared with oxygen cylinders. Oxygen concentrators were perceived as a modern technological solution to help patients. However, some caregivers in Lagos expressed being perplexed and concerned about their child when oxygen was delivered to patients via cylinder, as it is likened to the liquefied gas cylinders that most households in Lagos use for cooking.^44^ The misconception about oxygen cylinders was partly ascribed to the failure of HCPs to provide adequate counselling to caregivers prior to the commencement of oxygen treatment.

‘Also, the caregivers panic when they see cylinder for instance, we delivered triplet last month, we gave two (of the babies) oxygen via the concentrator, the third baby needed oxygen and we used cylinder. The mother and grandmother were scared as they thought there was complications, not knowing the other two have been on oxygen via concentrator’. HCP 3 Lagos

### The state of oxygen incoherence is modifiable

Knowledge of oxygen was a strong determinant of oxygen refusal or acceptance in both states. Oxygen access is comparatively better in Lagos than in Jigawa state^45^; thus, previous personal use of oxygen by caregivers or children (proximate knowledge) is common. Hence, it was not surprising that medical oxygen was considered a good intervention and not an unusual therapy. In contrast, awareness of oxygen was low in Jigawa and mostly related to use in adults with poor prognosis. This distal knowledge of oxygen was reported as a barrier to oxygen acceptance for children, as oxygen prescription for children was associated with an end-of-life event and hopelessness about survival, thus leading some caregivers to refuse medical oxygen treatment.

‘They would rather go, “let him die in peace”, that’s what they will tell you. Let the patient die in peace please, don’t (give oxygen)’. HCP 1 Jigawa

In both states, a lack of knowledge about the benefits of oxygen was identified as a reason for the caregiver’s refusal of medical oxygen for sick children.

Medical oxygen treatment was regarded as a ‘death sentence’, with survival for patients receiving oxygen not guaranteed. To others, oxygen is harmful, as it does not allow patients to breathe well. These misconceptions were described as pervasive community norms, which sometimes resulted in oxygen treatment refusal and/or emotional distress for caregivers—such as loss of hope for a child’s survival and fear of death.

‘I want to give an example, most times in movies you would see that patient who received oxygen treatment died in that movie, though I know that the death in movies is not true, but they don’t portray good image about oxygen treatment. So, my people here, the majority is illiterate; they can believe what they see in the movie and think it is real’. Participant 4, young women FGD, Jigawa

‘Some caregivers often think when a child needs oxygen in the hospital, it means the child can't survive the ailment. I was the one that calmed a woman down some weeks ago, she was scared and worried after she learnt her child would need oxygen. I told her to relax, that it’s just to assist the child, but she said she thought oxygen is used to revive a dead person or almost dead. I told her it’s to save the child’. Participant 2, Men FGD Imota Lagos

Despite the perception of medical oxygen as a death sentence, participants whose children received medical oxygen and died attributed the death to fate and not the oxygen. In contrast, scepticism, worries and fear associated with medical oxygen treatment often dissipate following a positive outcome from medical oxygen treatment for sick children.

‘Truly the story I heard about oxygen was, if they put oxygen for children, it’s used to be bad or the child may even die. So when I saw the nurses putting it on my baby, I felt bad and unhappy but I was surprised to see how her breathing pattern changed within few minutes because when we came to the hospital she was having difficulty in breathing’. Caregiver 2 Jigawa

Regarding the influence of the COVID-19 pandemic on oxygen perception, there was consensus among the participants in Jigawa that the pandemic did not affect caregiver’s refusal or acceptance of medical oxygen.

‘In my own opinion, the coming of COVID-19 did not change people’s perception of oxygen treatment’. Participant 6, MEN FGD Jigawa

In Lagos, which was the epicentre of the COVID-19 pandemic in Nigeria,^46^ there was a lack of consensus on the impact of the COVID-19 pandemic on medical oxygen acceptance. Medical oxygen was acknowledged to be important in the management of COVID-19 patients by the men’s group, and one HCP noted that the pandemic led to increased awareness of the importance of medical oxygen for patients with respiratory distress. However, other HCPs did not identify any impact of the pandemic on medical oxygen acceptance. There was no report of medical oxygen refusal because of its association with the COVID-19 infection.

### Opportunity cost of medical oxygen treatment

In Lagos, the fear of insolvency and a possible consequence being their child’s confinement to the hospital (ie, the child not being permitted to leave until the hospital bill is paid) was a strong barrier to oxygen acceptance among caregivers. Healthcare for children under the age of five is not fully subsidised in Lagos compared with Jigawa. As a result, out-of-pocket payments for healthcare can be high, especially when including fees for oxygen services. The huge financial implication of medical oxygen treatment was a major reason for oxygen refusal among caregivers, even among those who had earlier accepted medical oxygen for their children. Sometimes, caregivers disagree with the provider’s recommendation of oxygen therapy for their children because of the huge financial burden of oxygen therapy.

‘The bill per hour was high, she said there was no need for the oxygen therapy and that the child was fine’. HCP 4 Lagos

‘Money is the major reason (for refusing medical oxygen treatment) because she knows that if they should give the child the oxygen and the child gets better(smiles), she will pay for it. But if the child gets better and she has no money to pay, will she drop the child at the hospital? That’s why she will shout that, “don’t give my child oxygen please’. Lagos, Men FGD (urban), participant 5

Lagos participants highlighted the non-responsiveness of the health system to the health needs of the people, as illustrated in the quotes below:

‘Once they confirmed that oxygen is needed, they should give without wasting time. It should be life first, but the reverse is the case, (it is) money first!’ Lagos, MEN FGD (urban), participant 7

‘This applies to all General hospital, no money, no treatment, they don't care what the condition is’. Lagos, WOMEN FGD (Urban), Participant 13

In Jigawa, the cost of medical oxygen was not a barrier to caregivers’ acceptance of oxygen. Charging for medical oxygen was not a common practice in state-owned hospitals, and in addition, healthcare services were usually first provided before asking the caregivers to make payment after the child became stable. This practice dampens the impact of cost on oxygen acceptability, though participants alluded to financial barriers for other healthcare service utilisation.

‘In government hospitals like general hospital, oxygen is free. So, since it is free it shouldn’t be a problem when making the decision to accept the treatment’. Jigawa, Young Women FGD, participant 1

## Discussion

The aim of this study was to understand caregivers’ and community perceptions and experiences of medical oxygen and how these relate to the acceptance of recommended oxygen treatment for sick children in Nigeria. We found that medical oxygen treatment for children is associated with emotional and financial distress for caregivers, which is shaped by contextual factors such as the culture of care, the underlying burden of under-five mortality and fertility rates, healthcare financing structure, literacy levels and socioeconomic characteristics. Acceptance or refusal of oxygen treatment depends on how caregivers respond to the struggles, fear and other distress associated with medical oxygen provision. Lack of knowledge of oxygen was more apparent in Jigawa, and this provided opportunities for misconceptions about medical oxygen therapy for children to become established community perceptions. Despite greater awareness and experience of medical oxygen in Lagos, the opportunity cost, particularly fear of insolvency and the child’s confinement to the hospital after recovery, was a strong motivation for refusing oxygen treatment. The non-acceptance of oxygen treatment for sick children was modifiable, but the root causes in Lagos and Jigawa were different. Therefore, a one-size-fits-all policy will not work.

In line with the goal of universal health coverage as enshrined in the 2022 National Health Insurance Authority,^47^ making medical oxygen affordable to patients must be a central focus of all efforts aimed at improving oxygen access, particularly in countries with high out-of-pocket expenditures for healthcare.^48^ Studies have shown that oxygen costs can be high and may lead to catastrophic health expenditures.^49 50^ A study conducted by Graham *et al* in Lagos, before the COVID-19 pandemic, found that the median cost of medical oxygen for 2 days was US$36 and could be as high as US$267 in private facilities. ^51^ To make medical oxygen affordable to patients, there is a need to strengthen oxygen service delivery systems through inclusive collaboration, commitment and accountability among stakeholders, including patient groups and community members. In addition, we need to address the financing challenges associated with the oxygen service system in Nigeria. At the time of our study, oxygen-related planning, purchasing and management (including revenue raising) were mostly decentralised, with individual facilities typically required to mobilise funds from user fees—and sicker patients, therefore, bearing the greatest financial burden.

To optimise the impact of investment in medical oxygen systems and achieve equitable access for all patients, efforts to improve oxygen services must be integrated with efforts to strengthen other components of health systems. For example, in Jigawa state, oxygen refusal because of its associated financial burden was uncommon due to the practice of providing emergency care to patients before requesting money. This is in sharp contrast to the reports provided by caregivers and community members in Lagos, which has many public and private facilities but no effective policy of free healthcare for children—and therefore inhibitive out-of-pocket costs. Patients with emergency conditions need to have unconditional access to stabilisation services, irrespective of their financial status, to prevent avoidable mortalities.

Trust in the decisions made by HCPs plays an important role in oxygen acceptance, but it is impacted by HCP’s behaviours and relationships within the community they serve. Our finding of trust based on social relationships between HCPs and patients in Jigawa further strengthens the importance of empathy and good patient–provider interaction skills among HCPs. Though public trust transcends individual interactions with healthcare,^52^ HCPs need to see themselves as stakeholders in building public trust in healthcare. Negative behaviours of HCPs towards patients have been reported in multiple studies,^53^,^55^ partly due to systemic and contextual challenges that frustrate and demotivate HCPs.^53^ Simultaneously, violence and abuse towards HCPs leads to a negative and unsafe work and care environment. Therefore, building community trust and creating safe care environments are essential to improve service delivery.

In Jigawa, we did not find evidence from the discussions with caregivers that an HCP’s gender influences their acceptance of medical oxygen. This contrasts with findings from previous studies from Nigeria, which have reported gender preference for HCPs, particularly around vaccine service delivery and maternal healthcare.^56^,^60^ While there are strong gender norms in Jigawa,^55^ our finding may be due to the prescription of medical oxygen as a life-saving intervention during emergency clinical conditions in children compared with demand for vaccination or antenatal care services, which are non-emergency but essential healthcare services. Caregivers’ refusal of medical oxygen and willingness to allow the child to die in peace highlight the importance of culture and religion in healthcare service utilisation—particularly in northern Nigeria, where child death is typically perceived as an act of God.^61^ The involvement of religious leaders may be particularly important when introducing new or poorly understood health interventions and services, such as oxygen therapy.

Our findings around fear and the costs of oxygen resonate with findings in other sub-Saharan African settings. In Malawi, community members reported fear of oxygen as a treatment that causes death, which has also been noted, reflecting the dominant narrative of Jigawa more than Lagos.^17^ A study from Akure, South-West Nigeria, highlighted the challenge around the cost of oxygen, similar to our findings in Lagos.^26^ These findings differ from studies from high-income settings, which have reported physical burden and the inability to provide psychological support to their children as the main concerns of caregivers of children receiving oxygen therapy.^62 63^ Importantly, our findings suggest that, while negative beliefs and fears of oxygen therapy are present in contexts of high mortality and low oxygen service access, these narratives are also modifiable through improving access to and quality of care.

In our study, the recruitment of caregivers who had refused oxygen was a challenge as they did not remain at the facility. In Lagos, HCPs reflected that when oxygen is refused, this leads to a general withholding of further medical treatment by the facility and staff. This not only made it hard to find these participants for recruitment but also highlighted a point of tension between communities and HCPs. By refusing other potentially life-saving treatments, community trust around HCP motivations can be damaged. Similarly, HCPs can feel vulnerable to negative professional repercussions and violence from patient relatives if children under their care who are not receiving the recommended treatments do not survive. This contributes to their moral distress,^64 65^ which in turn can lead to demotivation, lack of job satisfaction, stress, compassion fatigue and poorer patient care.^66 67^

Our study has some key limitations. First, we did not explore the influence of oxygen delivery methods on caregiver’s perceptions of medical oxygen treatment. Given the narratives in Lagos around the use of cylinders, it may be that the mode of oxygen delivery could affect caregivers’ willingness to accept it. We had difficulty recruiting caregivers for interviews in Lagos, with many declining or cancelling interview appointments despite every effort by the research team. While we ultimately achieved sufficient quality interviews for saturation, it is possible that we might not have captured all the nuances of medical oxygen acceptance among caregivers in Lagos. Nevertheless, we have confidence in the findings, as the themes were relatively consistent and repeated across different respondents and aligned with previous literature. Second, we did not explore oxygen perception in acute surgical conditions as oxygen therapy is part of surgical/anaesthetic care and the caregiver would have provided consent as part of the procedural process. Third, we did not assess HCP’s knowledge of oxygen therapy and the quality of care provided, including the extra nursing care associated with oxygen therapy. It is possible that these factors may influence provider–client interaction and trust in healthcare around medical oxygen treatment and ultimately the caregiver’s decision to accept or refuse prescribed medical oxygen.

In conclusion, the community and caregiver’s acceptance of medical oxygen treatment is modifiable. Though the contextual barriers that manifest in fear are understandable, the root causes, however, differ in Lagos and Jigawa. Therefore, to improve caregiver’s acceptance of medical oxygen treatment, a one-size-fits-all policy is unlikely to work; rather, healthcare financing and provider-patient interaction must be strengthened through context-specific collaboration. In addition, there is a need for active community engagement to improve their understanding and attitude towards medical oxygen treatment.

## Supplementary material

10.1136/bmjgh-2023-014902 online supplemental file 1

## Data Availability

Data are available upon reasonable request.
