# Supplementary material for: ‘Let him die in peace’: understanding caregiver’s refusal of medical oxygen treatment for children in Nigeria
Source: BMJ Glob Health. 2024 May 16;9(5):e014902. doi: 10.1136/bmjgh-2023-014902 (PMC11103205; doi:10.1136/bmjgh-2023-014902)
Supplement: online supplemental file 1 [file bmjgh-2023-014902supp001.pdf]

Appendix 1: Health care provider oxygen experience interview topic guide

We will be conducting interviews with healthcare providers who work on neonatal and paediatric wards, which can provide oxygen and nasogastric feeding for children.

1. Clinical context
- Can you tell me about a typical day in your setting?
    - Probe: What sort of duties do you have on the ward? How many children do you see?
- Can you describe a typical case of pneumonia in your setting?
    - How long are they admitted for? What sort of treatments do they get?
- How do you decide if it is a severe or non-severe case of pneumonia?

2. Oxygen
- Can you tell me about the last time you provided oxygen to a child?
    - What was the child's illness? How long were they on it? Did they recover?
- How often do you provide oxygen?
- How do you decide who to give oxygen?
- Do you encounter any challenges in giving oxygen?
    - For example, do you have children who you cannot give it to? Or who refuse? Or cannot afford to have oxygen?
    - Do you run out of oxygen or have challenges with supply? What do you do when this happens?
- Do you ever need to refer a child for oxygen treatment?
    - Why did you need to refer them?
    - How do caregivers react? Do you encounter challenges?
    - Can you tell me about a case where this happened?
- In your experience, has this changed since COVID-19?
    - What? How? Why?

3. Feeding during illness
- Can you tell me how you normally manage feeding for children in this setting?
    - Is food provided by the facility? Do caregivers feed their children? Is this monitored by healthcare workers?
- Can you tell me about the last time you inserted a nasogastric tube for feeding in a child?
    - What was the child's illness? How long were they on it? Did they recover?
- How often do you provide nasogastric tubes?
- How do you decide who to give a nasogastric tube?
- Do you encounter any challenges in giving nasogastric tubes?
    - For example, do you have children who you cannot give it to? Or who refuse? Or cannot afford it?
- Do you encounter any other issues around child feeding?

4. Community perceptions

- How do caregivers react when you say their child needs oxygen?
- How do caregivers react when you say their child needs a nasogastric tube?
- What challenges do caregivers encounter feeding their sick child? How do they manage these?
    - Do they have to force feed? What are the reasons they do that?
- Do caregivers refuse these treatments?

    - Why?
- What about challenges with administering prescribed antibiotics for their children?
    - Have you encountered similar or different issues with caregivers for antibiotic treatment?
    - How do caregivers react when you administer or recommed IV treatments? Is this different from tablets/syrups?
    - Why do caregivers use antibiotics shorter than recommended period?
    - Why do caregivers use antibiotics longer than recommended period?

5. Recommendations

- What do you think is going right in your efforts to provide care for severely sick children? What are the main enablers of these efforts?
- What are the main barriers you face in providing care for severely sick children?
-

Appendix 2: Caregiver oxygen experience interview topic guide

Thanks for agreeing to talk to me today. The point of the interview is to understand your experiences your child's recent illness. First, I want to get to know a bit more about you and your typical family life. Can you tell me what an average day is like? What do you do? (Ice breaking question- also gives wider context)

1. Tell me a little bit about yourself and family.

- How many children do you have?
- What is your relationship with the father/mother of your children? (What kind of marriage?)
- Who lives in your household?
- Where were you born? Are you from here?

2. Care-seeking

Tell me the story of your child's recent illness, including the care you received from any providers and the decisions you/your family/a healthcare provider made.

- Can you tell me about how you recognised that your child was ill and decided to seek care?
- What made you think your child was sick? What symptoms did you recognize?
- After you decided your child was sick, where and when was the decision to seek care made? Does anything about the child change this decision (e.g. gender/age)?
- Who made the decision to seek care, you, your partner, other family members? together? how do you usually make decisions about seeking health care in your family?
- Did your communities cultural beliefs or religion influence your decision? In what way?
- Do your own cultural beliefs or religion influence where you decided to seek care? In what way?
- Why did you decide to go there? Was this your first choice?
- How did you travel there, how long did it take and cost?

3. Inpatient care

- How long did it take to get to the hospital? How did you get there?
- What type of hospital was it (e.g. private/mission)? Why did you choose to go to this facility?
- What happened when you got to the hospital?
- What care did they receive when they were admitted?
- How much did this care cost? How long was your child admitted for?
- Did you leave against medical advice? Why?

4. Oxygen treatment

- Was your child recommended for oxygen treatment?
- How long did your child receive oxygen for? Was this the recommended time, or shorter/longer?
- How did you decide for your child to receive this treatment? Were you hesitant? Why?
- Did the healthcare provide explain this treatment to you?
- Did you pay for this treatment? Were you willing to pay? How much?
- Had you heard about oxygen treatment before you came here? What did you know about it?

5. Feeding

- Did you face challenges with feeding your child while being sick? How did you manage it?

- Did you use force feeding an alternative approach if your child is not eating well? Why did you decide to do this?
- Was your child recommended for tube feeding?
- How long did your child receive this feeding for? Was this the recommended time, or shorter/longer?
- How did you decide for your child to receive this treatment? Were you hesitant? Why?
- Did the healthcare provide explain this treatment to you?
- Did you pay for this treatment? Were you willing to pay? How much?
- Had you heard about tube feeding before you came here? What did you know about it?
- Did your child receive any other food or drink while they had this tube? Why was that given?

Appendix 3: Focus group discussion interview topic guide

Introduction

Good morning sir/ma, my name is XX. I work in partnership with Save the Children Nigeria/University College Hospital Ibadan. Today, we want to learn from you about care for sick children. We want to know how you cope with challenges associated with sick children, particularly feeding practices, antibiotics use, care seeking, and referral advice by healthcare workers. When children are sick, particularly those with pneumonia, they may require oxygen therapy, nasogastric feeding, and referral to a higher level of care. We want to know your perceptions about each of these, and how they are handled in this setting. We will tell you practical case examples of care experiences for sick under-five children. We will pause to ask about your thoughts. Is that ok?

1. Aishat is a 2-year-old girl with fever and cough in the last 2 weeks. Her father has traveled to a nearby state for work. Aishat's mother prefers traditional medicine to medical care. She went to a native doctor(Jigawa) /Alagbo/elewe omo (Lagos) for treatment and has been applying the herbal medicine for her child but no improvement. Aishat's condition was getting critical and her mother was advised to take Aishat to a nearby PHC. On getting there, the healthcare provider assessed the child and informed Aishat's mother that she will need inpatient care, as Aishat is very sick. She referred Aishat to Dutse General Hospital(Jigawa)/Ikorodu General Hospital(Lagos)

    a. Your general opinion about (i) treatments received at home by Aishat and (2) Aishat's mother's preference for traditional care over medical care—how common is this? Why? What roles do traditional healers play in child health?

    b. In this case, what challenges might the family face in going to the hospital?
    c. What factors would influence the decision to go?
        i. Probe healthcare provider attitude
        ii. Probe transportation means
        iii. Probe reports/rumours that people die there
        iv. Probe cost
    d. What would you do in this situation if you were Aishat's parents/grandparents? Why?

2. Nuhu is 1 year old admitted to the hospital for severe pneumonia. The healthcare provider informed Nuhu's mother that he needs oxygen therapy.

    a. Are you familiar with oxygen? What do you understand about this treatment?
        i. Probe if it implies (i) severe illness, (ii) death sentence (iii) cause for worry/anxiety
    b. In this case, what challenges might the family face in accepting oxygen treatment?
    c. What factors would influence the decision to accept or not accept the treatment? Probe cost, past experience of oxygen therapy, Nollywood movies, and if HCW explains treatment to caregivers
    d. What would you do in this situation if you were Nuhu's parents/grandparents? Why?
    e. Has this changed since COVID-19? Why?

3. Abdullahi is 3 years old and has been sick for the past 10 days with fever, vomiting, diarrhea and cough. Abdullahi is admitted to the hospital and the healthcare provider has informed Abdullahi's parents that he requires feeding through a tube.

    a. Are you familiar with feeding through a tube? What do you understand about this treatment?
    b. In this case, what challenges might the family face in accepting tube feeding treatment?
    a. What factors would influence the decision to accept or not accept the treatment? Probe cost, past experience of nasogastric feeding, Nollywood movies, and if HCW explains treatment to caregivers
    c. What would you do in this situation if you were Abdullahi's parents/grandparents? Why?

4. A. Jigawa Narrative

Mallam Danladi is a rich farmer with 2 wives and 10 children. He has 30 cows, 10 camels, and 3 poultry farms, each with at least 100 chickens (layers). Despite Mallam's Danladi's wealth, four of his children, however, are malnourished—two are on admission for nutritional therapy.

a. Are you familiar with cases like Mallam Danladi in your community?

Mallam Danladi believes his wives are not caring enough for the children well that is why they are sick. He said sometimes his wives will eat 6-7 times daily without being bothered if their child refuses to eat.

b. Do you agree with Mallam Danladi? Have you observed similar things in your community? Are there people in your community with similar belief to Mallam Danladi?

Another child to the second wife is sick and he refuses to eat. In other to ensure he takes something, the second wife forcefully feeds the child with pap (closing his nostrils and forcefully opening his mouth)

c. Are you familiar with force-feeding practices? What are these? How common are these? What is your perception about this? When or why do they happen? Who usually initiates this? Grandparent? Older wives or community members?
d. Have you ever faced challenges with feeding a child who is sick? How did you manage? Have you ever faced challenges with giving medications to a child who is sick? How did you manage?

B. Lagos Narrative

Mr. Ajibutu is a rich businessman. He sells car spare parts with shops in Ikorodu, Oshodi, Owode and Ladipo markets. Despite Mr. Ajibutu's wealth, four of his children, however, are malnourished—two are on admission for nutritional therapy.

a. Are you familiar with cases like Mr Ajibutu in your community?

Mr Ajibutu believes his wives are not caring enough for the children well that is why they are sick. He said sometimes his wives will eat 6-7 times daily without being bothered if their child refuses to eat.

b. Do you agree with Mr Ajibutu? Have you observed similar things in your community? Are there people in your community with similar belief to Mr Ajibutu?

Another child to the second wife is sick and he refuses to eat. In other to ensure he takes something, the second wife forcefully feeds the child with pap (closing his nostrils and forcefully opening his mouth)

c. Are you familiar with force-feeding practices? What are these? How common are these? What is your perception about this? When or why do they happen? Who usually initiates this? Grandparent? Older wives or community members?
d. Have you ever faced challenges with feeding a child who is sick? How did you manage? Have you ever faced challenges with giving medications to a child who is sick? How did you manage?

5. Dauda is sick with cough and fever. Her mother takes him to a nearby PHC and he's prescribed amoxicillin for 5 days. After 3 days of use, Dauda's mother no longer gives Dauda the medication again.
    a. How common is this in your community?
    b. In this case, what challenges do caregivers encounter with antibiotics use in terms of procurement, use and storage?
    c. What make caregivers use antibiotics for their children for shorter duration than the recommended time? Is that common here?
    d. What make caregivers use antibiotics longer than the prescribed duration for their children? Is that common here?
    e. Do caregivers mix traditional medicine with orthodox medicine? How common? and why?
